# Supplementary material for: Multiphoton non-local quantum interference controlled by an undetected photon
Source: Nat Commun. 2023 Mar 17;14:1480. doi: 10.1038/s41467-023-37228-y (PMC10023773; doi:10.1038/s41467-023-37228-y)
Supplement: Supplementary file 1 — Supplementary Information [file 41467_2023_37228_MOESM1_ESM.pdf]

Supplementary Information for "Multiphoton non-local quantum interference controlled by an undetected photon"

Kaiyi Qian,[1,*] Kai Wang,[1,†] Leizhen Chen,[1] Zhaohua Hou,[1] Mario Krenn,[2,‡] Shining Zhu,[1] and Xiao-Song Ma[1,3,4,§]

[1]*National Laboratory of Solid-state Microstructures, School of Physics, Collaborative Innovation Center of Advanced Microstructures, Nanjing University, Nanjing 210093, China*
[2]*Max Planck Institute for the Science of Light (MPL), Erlangen, Germany*
[3]*Synergetic Innovation Center of Quantum Information and Quantum Physics, University of Science and Technology of China, Hefei, Anhui 230026, China*
[4]*Hefei National Laboratory, Hefei 230088, China*

(Dated: March 4, 2023)

SUPPLEMENTARY NOTE

Supplementary Note 1 – Four-photon frustrated interference

In this section, we give the equations used in the main text. We consider the Hamiltonian of spontaneous parametric down-conversion(SPDC)

$$\hat{H} = i\eta(\hat{p}\hat{a}^\dagger\hat{b}^\dagger - p^\dagger\hat{a}\hat{b}) \tag{1}$$

as $\eta \ll 1$, we use the approximate transformation of SPDC:

$$\begin{aligned} U_{\mathrm{SPDC}} = e^{-i\hat{H}t} &= \hat{I} + g(\hat{p}\hat{a}^\dagger\hat{b}^\dagger - p^\dagger\hat{a}\hat{b}) + O(g^2) \\ &\approx \hat{I} + g(\hat{p}\hat{a}^\dagger\hat{b}^\dagger - p^\dagger\hat{a}\hat{b}), \end{aligned} \tag{2}$$

where $g = \eta t$. Here we omit the two-pair generation process in the same crystal, as the coincidence would be covered by the high intensity of single pair (shown below). With Supplementary Eq.(2), The state after crystals I and II are

$$\begin{aligned} |\psi_1\rangle &= U_{\mathrm{II}}U_{\mathrm{I}}|\alpha,\alpha,vac\rangle \\ &= [\hat{I} + g\alpha\hat{a_1}^\dagger\hat{a_4}^\dagger]_{\mathrm{II}}[\hat{I} + g\alpha\hat{a_2}^\dagger\hat{a_3}^\dagger]_{\mathrm{I}}|\alpha,\alpha,vac\rangle \end{aligned} \tag{3}$$

where $|\alpha,\alpha,vac\rangle$ represents the initial two coherent pump states and the vacuum in the down-converted modes. The pump, signals, and idlers will experience different phase shifts:

$$\begin{aligned} |\psi_2\rangle &= U^{\phi_{s1}}U^{\phi_{i1}}U^{\phi_{s2}}U^{\phi_{i2}}U^{\phi_{p1}}U^{\phi_{p2}}|\psi_1\rangle \\ &= [\hat{I} + g\alpha e^{i(\phi_{i2}+\phi_{s2})}\hat{a_1}^\dagger\hat{a_4}^\dagger]_{\mathrm{II}}[\hat{I} + g\alpha e^{i(\phi_{i1}+\phi_{s1})}\hat{a_2}^\dagger\hat{a_3}^\dagger]_{\mathrm{I}}|\alpha e^{i\phi_{p1}},\alpha e^{i\phi_{p2}},vac\rangle \end{aligned} \tag{4}$$

where $U^\phi = e^{i\phi a^\dagger a}$. After crystals III and IV, the final state is:

$$\begin{aligned} |\psi_f\rangle =& U_{\mathrm{III}}U_{\mathrm{IV}}|\psi_2\rangle \\ =& [\hat{I} + g(\alpha e^{i\phi_{p1}}\hat{a_1}^\dagger\hat{a_2}^\dagger - \hat{p_1}^\dagger\hat{a_1}\hat{a_2})]_{\mathrm{III}}[\hat{I} + g(\alpha e^{i\phi_{p2}}\hat{a_3}^\dagger\hat{a_4}^\dagger - \hat{p_2}^\dagger\hat{a_3}\hat{a_4})]_{\mathrm{IV}} \\ & [\hat{I} + g\alpha e^{i(\phi_{i2}+\phi_{s2})}\hat{a_1}^\dagger\hat{a_4}^\dagger]_{\mathrm{II}}[\hat{I} + g\alpha e^{i(\phi_{i1}+\phi_{s1})}\hat{a_2}^\dagger\hat{a_3}^\dagger]_{\mathrm{I}}|\alpha e^{i\phi_{p1}},\alpha e^{i\phi_{p2}},vac\rangle \end{aligned} \tag{5}$$

Again, we omit the terms with $O(g^2)$ and get

$$\begin{aligned} |\psi_f\rangle =& \hat{I} + g\alpha e^{i\phi_{p1}}\hat{a_1}^\dagger\hat{a_2}^\dagger + g\alpha e^{i\phi_{p2}}\hat{a_3}^\dagger\hat{a_4}^\dagger + g\alpha e^{i(\phi_{i2}+\phi_{s2})}\hat{a_1}^\dagger\hat{a_4}^\dagger + g\alpha e^{i(\phi_{i1}+\phi_{s1})}\hat{a_2}^\dagger\hat{a_3}^\dagger \\ &+ g^2\alpha^2[e^{i(\phi_{i1}+\phi_{s1}+\phi_{i2}+\phi_{s2})}\hat{a_1}^\dagger\hat{a_2}^\dagger\hat{a_3}^\dagger\hat{a_4}^\dagger + e^{i(\phi_{p1}+\phi_{p2})}\hat{a_1}^\dagger\hat{a_2}^\dagger\hat{a_3}^\dagger\hat{a_4}^\dagger \\ &+ e^{i(\phi_{p1}+\phi_{i2}+\phi_{s2})}(\hat{a_1}^\dagger)^2\hat{a_2}^\dagger\hat{a_4}^\dagger + e^{i(\phi_{p1}+\phi_{i1}+\phi_{s1})}\hat{a_1}^\dagger(\hat{a_2}^\dagger)^2\hat{a_3}^\dagger \\ &+ e^{i(\phi_{p2}+\phi_{i2}+\phi_{s2})}\hat{a_1}^\dagger\hat{a_3}^\dagger(\hat{a_4}^\dagger)^2 + e^{i(\phi_{p2}+\phi_{i1}+\phi_{s1})}\hat{a_2}^\dagger(\hat{a_3}^\dagger)^2\hat{a_4}^\dagger]|\alpha e^{i\phi_{p1}},\alpha e^{i\phi_{p2}},vac\rangle. \end{aligned} \tag{6}$$

Supplementary Eq.(6) could be rewritten as the photon number state of spatial modes 1-4:

$$\begin{aligned}|\psi\rangle =&|\alpha e^{i\phi_{p1}}, \alpha e^{i\phi_{p2}}\rangle_{p_1,p_2} \otimes \{|vac\rangle + p[e^{i(\phi_{s1}+\phi_{i1})}|0110\rangle + e^{i(\phi_{s2}+\phi_{i2})}|1001\rangle + e^{i\phi_{p1}}|1100\rangle + e^{i\phi_{p2}}|0011\rangle] \\ &+ p^2[e^{i(\phi_{i1}+\phi_{s1}+\phi_{i2}+\phi_{s2})}|1111\rangle + e^{i(\phi_{p1}+\phi_{p2})}|1111\rangle \\ &+ \sqrt{2}e^{i(\phi_{i1}+\phi_{s1}+\phi_{p1})}|1210\rangle + \sqrt{2}e^{i(\phi_{i1}+\phi_{s1}+\phi_{p2})}|0121\rangle + \sqrt{2}e^{i(\phi_{p1}+\phi_{i2}+\phi_{s2})}|2101\rangle + \sqrt{2}e^{i(\phi_{p2}+\phi_{i2}+\phi_{s2})}|1012\rangle]\}.\end{aligned} \tag{7}$$

When we consider only the down-converted photons and set $\phi_{p1} = \phi_{p2}$, Supplementary Eq.(7) is exactly Eq.(1).

With Eq.(1), the terms that contribute to coincidence of 1, 3, 4 is $p^2e^{i(\phi_{i1}+\phi_{s1}+\phi_{i2}+\phi_{s2})}|1111\rangle$, $p^2e^{i2\phi_p}|1111\rangle$, $\sqrt{2}p^2e^{i(\phi_p+\phi_{i2}+\phi_{s2})}|1012\rangle$. The first two terms interfere and the last term contributes a constant noise. Therefore, the coincidence rate is

$$P_{1,3,4} = p^4|e^{i(\phi_{i1}+\phi_{s1}+\phi_{i2}+\phi_{s2})} + e^{i2\phi_p}|^2 + 2p^4 = p^4[4 + 2\cos(\phi_{i1} + \phi_{s1} + \phi_{i2} + \phi_{s2} - 2\phi_p)] \tag{8}$$

Supplementary Note 2 – Two-photon frustrated interference

To show the four-photon interference, we first build two identical individual interferometers between sources I and III, and II and IV. In Supplementary Fig. 1, we show the interferometer between I and III. The scheme is almost the same as in Fig. 2b, except for the QWP in the Swap module, which is fixed at 0° instead of 45° so that i1 and i2 do not exchange their path.

To test the interference of sources I and III, we block the pump P2, fix the position of M2 (ϕ_p), and perform a coarse scan of the phases of down-converted photons (ϕ_i, ϕ_{s1}) until the interference fringe of two sources emerges. As shown in Supplementary Fig. 2(a), the envelope indicates about 0.2-mm coherent length of the down-converted photons. Then we fix M3 (ϕ_{s1}) at the place where the visibility of interference is maximum and finely tune the phase ϕ_i to obtain the interference pattern. The result is shown in Supplementary Fig. 2(b). The visibility of two-photon coincidence is 95.5%. For sources II and IV, we carry out the same operations. The result is shown in Supplementary Fig. 2(c) with a visibility of 95.0%. The error bar is smaller than the data point and is not shown here.

The high visibility of two-photon frustrated interference (FI) ensures the path identity, which is essential for observing the interference of four sources. We also observe single-photon FI with high visibilities, showing high-level indistinguishability of the photons on the same path, as shown in Supplementary Fig. 3(a), (b).

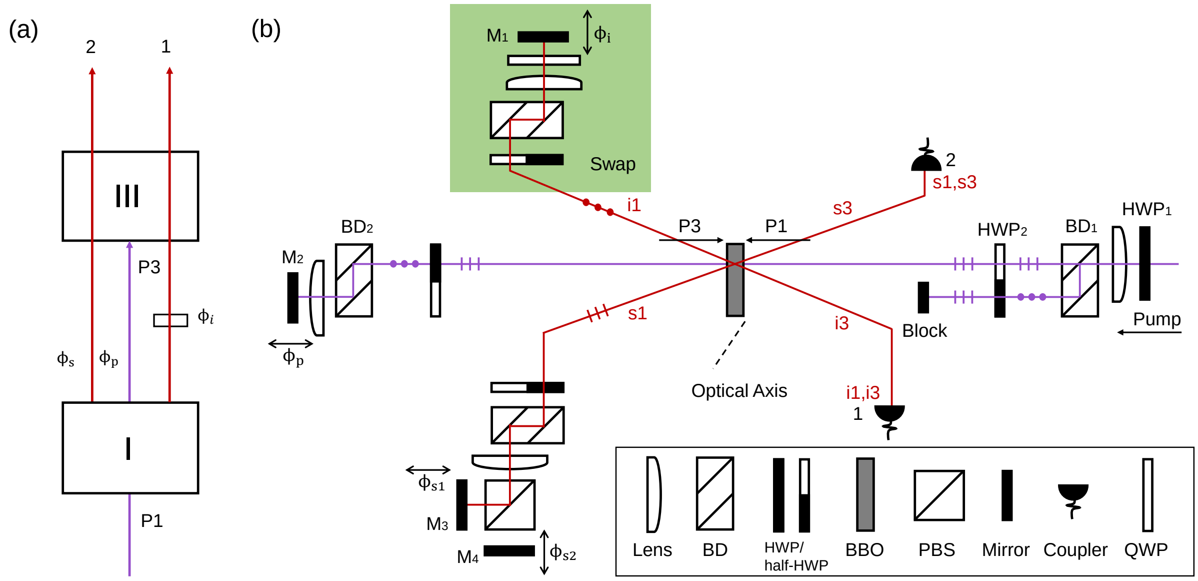

Supplementary Fig. 1. (a) Scheme of frustrated two-photon generation (sources I and III). One pump light pumps two crystals placed in sequence. The signal and idler photons from different sources are aligned to ensure the path identity. The counts of single-photon and two-photon coincidence depend on the phases of pump and down-converted photons: $I \propto 1+\cos(\phi_i+\phi_s-\phi_p)$. (b) Experiment setup of the interferometer (sources I and III). We block P2 in Fig. 2b and fix the angle of QWP at 0° to study the FI of photons from P1 and P3.

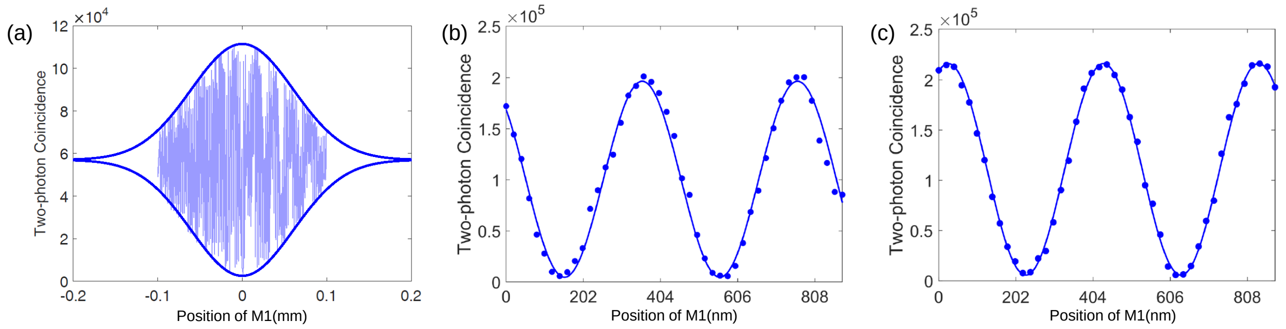

Supplementary Fig. 2. (a) Coarse scan of the phase ϕ_i helps to find the locations of interference fringes. (b)/(c) Results of two-fold coincidence counts for the frustrated interference from sources I, III/sources II, IV. The horizontal axis is the position of mirror M1. The interference visibility is (b)/(c) 95.5%/95.0%. The integration time of each point is 2 s.

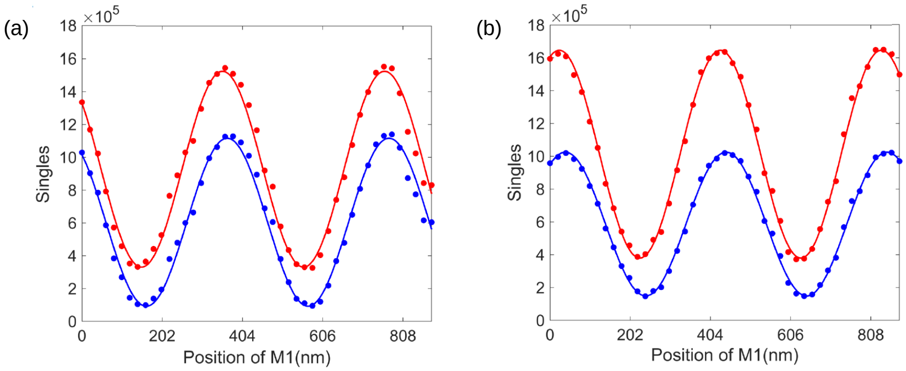

Supplementary Fig. 3. Single-photon counts for the frustrated interference from (a) sources I, III and (b) sources II, IV. The horizontal axis is the position of mirror M1. (a) Blue/red points represent the single counts on detector 1/2 and the visibility of the fitting curve is 84.7%/64.3% (b) Blue/red points represent the single counts on detector 3/4 and the visibility of the fitting curve is 74.6%/62.5%. The integration time of each point is 2 s.

Supplementary Note 3 – Spatial alignment and interference visibility

In this section, we discuss the causes of the reduced visibility of FI. We start from the two-photon FI discussed above. The misalignment of photons on the same path (i1/s1 and i3/s3) and the additional loss for photons i1 and s1 on the optical elements give rise to the different coupling efficiency for sources I and III. Therefore, the collected intensity of source I is lower than that of source III, which is the main reason for the limited visibility. This can be seen in Supplementary Table 1, where the two-fold coincidence counts of sources I and II are lower than that of sources III and IV. If we model the different intensity as transmissivity T, the quantum state of sources I and III is

$$|\psi\rangle_{1,3} = p[T_1|11\rangle_{i_1s_1} + R_1|11\rangle_{i_1's_1'} + |11\rangle_{i_3s_3}], \tag{9}$$

where T_1 is the transmissivity and R_1 is the reflectivity of source I. Photons in the second term are dissipated and not detected. $|11\rangle_{i_1s_1}$ and $|11\rangle_{i_3s_3}$ are indistinguishable and will interfere. The visibility of Supplementary Eq.(9) is $V = \frac{2T_1}{1+T_1^2}$, from which we can estimate T_1 is about 0.737 with $V = 95.5\%$. For sources II and IV, we have a similar analysis and get $T_2 = 0.724$ with $V = 95.0\%$.

For the four-photon FI, as the beam spacing and parallelism from the BDs are different, the coupling efficiencies of i1 and i2 reduce significantly after the swapping, which aggravates the intensity imbalance. Therefore, we have to make a compromise between the different sources. The result is shown in Supplementary Table 1. q_i is the ratio of intensity after and before the swapping of source i. Considering that both T_1 and T_2 above will furthermore reduce the visibility of four-fold coincidence, the quantum state of four-photon FI is

$$|\psi\rangle_{1,2,3,4} = p^2[\sqrt{q_1q_2}T_1T_2e^{i(\phi_{i1}+\phi_{s1}+\phi_{i2}+\phi_{s2})}|1111\rangle_{i_2s_1i_1s_2} + \sqrt{q_3q_4}e^{i2\phi_p}|1111\rangle_{i_3s_3i_4s_4}], \tag{10}$$

where the terms that do not contribute to the four-fold coincidence are omitted, and only the two terms that interfere remain. The visibility of Supplementary Eq. (10) is $V = \frac{2\alpha}{1+\alpha^2} = 81.9\%$, where $\alpha = \sqrt{\frac{q_1q_2}{q_3q_4}}T_1T_2 = 0.521$. This

	Coincidence (signal,idler)$\times 10^4/s$		
	Before (N_1)	After (N_2)	$q_i = N_2/N_1$
Source I	1.75	1.03	0.589
Source II	2.17	2.16	0.995
Source III	3.27	2.43	0.743
Source IV	2.83	2.34	0.827

Supplementary Table 1. Two-fold coincidence counts of the four sources before and after the swapping of i1 and i2.

	Coincidence (signal,idler)$\times 10^4/s$		
	Before (N_1)	After (N_2)	$q_i = N_2/N_1$
Source I	1.30	0.53	0.408
Source II	1.21	1.41	1.17
Source III	2.13	2.14	1.005
Source IV	2.24	1.79	0.799

Supplementary Table 2. Two-fold coincidence counts of the four sources with lower α.

estimated visibility from independent two-fold coincidence counts is close to the experiment result of 75.47% as in the main text. We estimate the reduction of visibility from higher order emission is about 10% from independent measurement.

For comparison, we reduce α to 0.411, as shown in Supplementary Table 2 and measured the interference pattern for four-photon coincidence counts again. We find the four-photon interference visibility decreases to 67.2% as shown in Supplementary Fig. 4. The result is consistent with our theoretical prediction $V = 70.32\%$ with $\alpha = 0.411$.

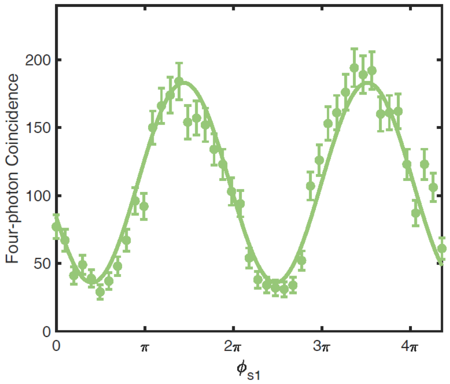

Supplementary Fig. 4. Result of four-fold coincidence counts. The horizontal axis represents the position of M3 (ϕ_{s1}). The fitting curve has visibility of 67.2% and a period of 418.9 nm. The error of visibility calculated from Poisson statistics is 4.50%.

Supplementary Note 4 – Time control of four-photon interference

The temporal indistinguishability for photons on the same path is essential for the four-photon interference[1]. In this section, we analyze the time of the photons generated from different crystals. The lengths of important parts are labeled in Supplementary Fig. 5.

We start our discussion from the two-photon interference scheme, where the QWP is fixed at 0°. The interference of sources I and II occurs when the pump P3 and the photons s1, i1 arrive at the BBO crystal simultaneously. That's

$$t_{i1} = t_{s1} = t_{P3} \tag{11}$$

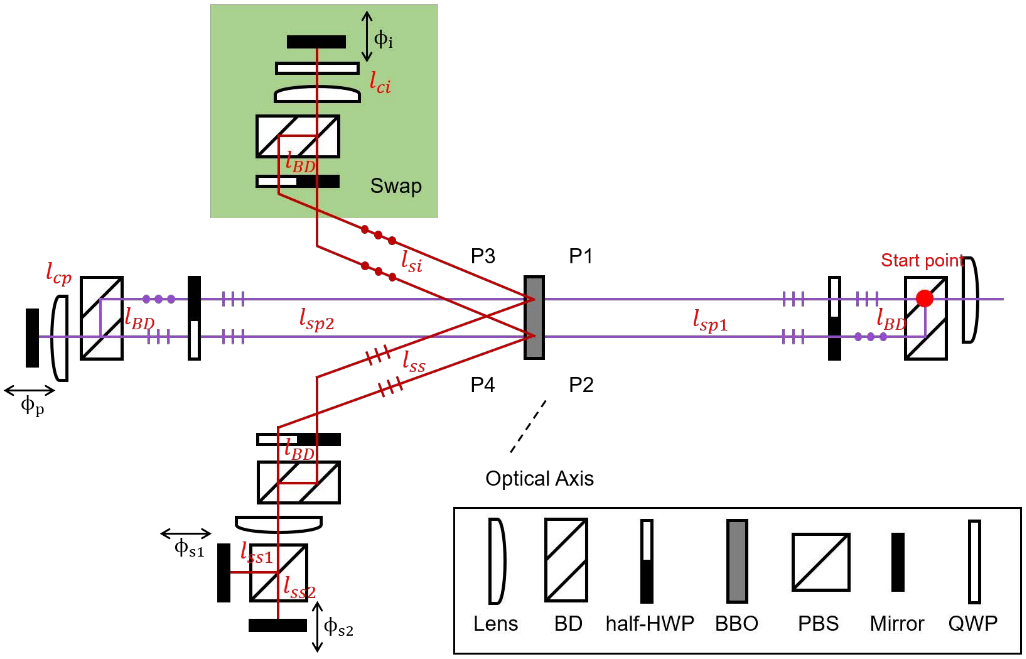

Supplementary Fig. 5. Experiment setup. The lengths of different optical paths are labeled in red.

where

$$
\begin{aligned}
t_{i1} &= \frac{1}{c}(l_{sp1} + l_{si} + l_{BD} + l_{ci} + l_{ci} + l_{BD} + l_{si}) \\
&= \frac{1}{c}(l_{sp1} + 2l_{si} + 2l_{ci} + 2l_{BD}) \qquad (12) \\
t_{s1} &= \frac{1}{c}(l_{sp1} + l_{ss} + l_{BD} + l_{ss1} + l_{ss1} + l_{BD} + l_{ss}) \\
&= \frac{1}{c}(l_{sp1} + 2l_{ss} + 2l_{ss1} + 2l_{BD}) \qquad (13) \\
t_{P3} &= \frac{1}{c}(l_{sp1} + l_{sp2} + l_{BD} + l_{cp} + l_{cp} + l_{BD} + l_{sp2}) \\
&= \frac{1}{c}(l_{sp1} + 2l_{sp2} + 2l_{cp} + 2l_{BD}) \qquad (14)
\end{aligned}
$$

represent the time experienced by the photons i1, s1, and pump P3, respectively. The beginning point of time is when the pump incident onto BD1, where the pump splits into two paths, as denoted in Supplementary Fig. 5 with “start point”. As the position of M2 is fixed ($l_{sp2} + l_{cp}$), Supplementary Eq. (11) could be rewritten as

$$t_{i1} = t_{P3} \Rightarrow l_{ci} = l_{sp2} + l_{cp} - l_{si} \qquad (15)$$
$$t_{s1} = t_{P3} \Rightarrow l_{ss1} = l_{sp2} + l_{cp} - l_{ss} \qquad (16)$$

Thus, we should adjust M1 and M3 to meet the above conditions. For sources II and IV, we have similar conditions:

$$l_{ci} = l_{sp2} + l_{cp} - l_{si} \qquad (17)$$
$$l_{ss2} = l_{sp2} + l_{cp} - l_{ss} \qquad (18)$$

When we swap i1 and i2, the time the photons experience are as follows:

$$t'_{i1} = \frac{1}{c}(l_{sp1} + 2l_{si} + 2l_{ci} + l_{BD}) \tag{19}$$

$$t_{s1} = \frac{1}{c}(l_{sp1} + 2l_{ss} + 2l_{ss1} + 2l_{BD}) \tag{20}$$

$$t'_{i2} = \frac{1}{c}(l_{sp1} + 2l_{si} + 2l_{ci} + 2l_{BD}) \tag{21}$$

$$t_{s2} = \frac{1}{c}(l_{sp1} + 2l_{ss} + 2l_{ss2} + l_{BD}) \tag{22}$$

$$t_{P3} = \frac{1}{c}(l_{sp1} + 2l_{sp2} + 2l_{cp} + 2l_{BD}) \tag{23}$$

$$t_{P4} = \frac{1}{c}(l_{sp1} + 2l_{sp2} + 2l_{cp} + l_{BD}) \tag{24}$$

As long as the conditions of Supplementary Eq. (15) - (18) satisfies, which could be realized by keeping M2, M3, M4 unchanged and scanning the position of M1, the following equations of time indistinguishability still hold:

$$t'_{i2} = t_{P3} \text{ (path1)}; \quad t_{s1} = t_{P3} \text{ (path2)}; \tag{25}$$

$$t'_{i1} = t_{P4} \text{ (path3)}; \quad t_{s2} = t_{P4} \text{ (path4)}; \tag{26}$$

The above analysis shows that, though the photons from the sources I to IV are generated asynchronously[1], we still can't distinguish the photon on the same path by time, which is essential for the four-photon FI.

Supplementary Note 5 – Two-fold coincidence counts in the four-photon frustrated interference

We also analyze the two-fold coincidence counts in the four-photon interference experiment. The result is shown in Supplementary Fig. 6. There are six two-fold coincidences of the four photons on modes 1 to 4. Only the coincidences on detectors 1 and 3 or detectors 2 and 4 will show the interference pattern, as shown in Supplementary Fig. 6(a). The interference is a result of four-photon FI. Affected by the noise from the first order and second order, as shown in Eq. (1), the interference visibility is limited. The coincidence counts of detectors 2, 3 (source I), detectors 1, 4 (source II), detectors 1, 2 (source III) and detectors 3, 4 (source IV) show no interference as shown in Supplementary Fig. 6(b).

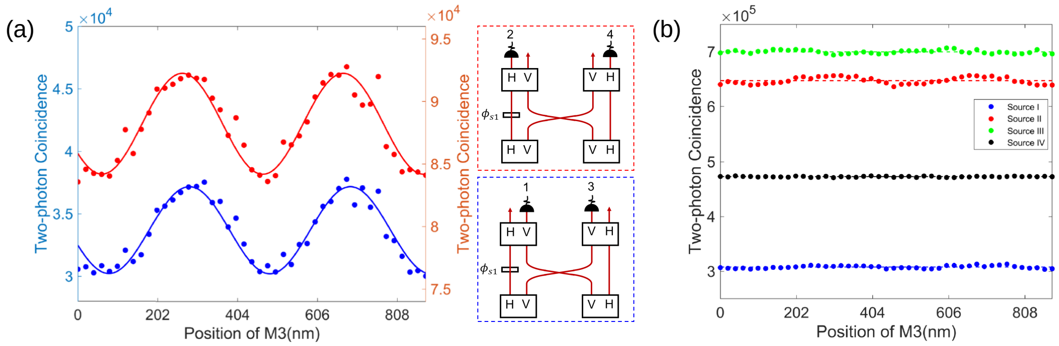

Supplementary Fig. 6. Results of two-fold coincidence counts in the four-photon frustrated interference. The horizontal axis represents the position of M3. (a) The red line represents the coincidence of detectors 2 and 4. The fitting curve has a visibility of 4.57% and a period of 407.0 nm. The blue line represents the coincidence of detectors 1 and 3. The fitting curve has a visibility of 10.33% and a period of 407.2 nm. (b) The coincidence counts of photons from four different sources I (detector 2,3), II (detector 1,4), III (detector 1,2), and IV (detector 3,4) show no interference, as expected.

Supplementary Note 6 – Visibility and transmissivity (V-T)

In this section, we give the function used for fitting the V-T correlation in the main text. Supplementary Eq. (10) gives the final state collected by detectors 1-4. When we reduce the transmissivity T of photon s2, it should be

rewritten as:

$$|\psi\rangle_{1,2,3,4} = \sqrt{q_3 q_4} p^2 [\alpha T e^{i(\phi_{i1}+\phi_{s1}+\phi_{i2}+\phi_{s2})} |1111\rangle_{i_3 s_3 i_4 s_4} + \alpha R e^{i(\phi_{i1}+\phi_{s1}+\phi_{i2}+\phi_{s2})} |1111\rangle_{i_3 s_3 i_4 s_2} + e^{i2\phi_p} |1111\rangle_{i_3 s_3 i_4 s_4}], \tag{27}$$

where we have denoted identical photons with the same subscript. R is the reflectivity of photon s2. The second term is dissipated and not detected. Therefore, the second term will not contribute to four-fold coincidence but to coincidences on detectors 1, 2, and 3. The visibility of the above equation for four-fold coincidence is

$$V = \frac{2\alpha T}{1 + (\alpha T)^2} \tag{28}$$

We consider α as a variable and are used to quantify the path identity of the photons or the intensity imbalance of the sources, as stated above. Supplementary Fig. 7 shows the four-photon FI with different transmissivities of photon s2, which corresponds to Fig. 3c. As T is reduced, the visibility tends to vanish. The α calculated from coincidence counts (0.521) is higher than that from the fitting curve (0.42) with Supplementary Eq. (28) . The difference may come from the limited long-time stability of our experiment and multiphoton noise from high-order emission.

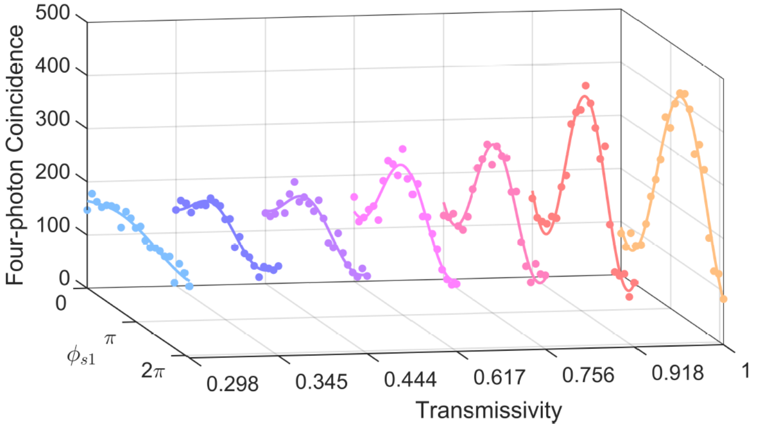

Supplementary Fig. 7. The relationship between the visibility of four-photon coincidence and the transmissivity of photon s2.

For the coincidences on detectors 1, 2, and 3, as the second term in Supplementary Eq. (27) and term $|1210\rangle$ in Eq. (1) will contribute a constant noise, the visibility is

$$V = \frac{2\alpha T}{\alpha^2 + 3}. \tag{29}$$

V is proportion to T. Supplementary Fig. 8 shows the four-photon FI with different transmissivities of photon s2, which corresponds to Fig. 4c. As T is reduced, the visibility again tends to vanish.

Supplementary Note 7 – Space-time diagram of non-local and local quantum interference

Throughout our manuscript, we use the terms 'non-local' by its operational definitions. Following the strict Einstein's locality conditions, when we say events X and Y are non-local, it means X lies outside of both the future and past lightcones of Y.

The quantum interference in the frustrated two-photon generation is the interference between photon sources from two crystals. Based on the above definition of non-local, as the twin photons are generated in the same place, the phase setting event **a** is always in the past light cone of the detection event **B** (see Supplementary Fig. 9b below). Therefore, we call it local quantum interference.

The analogy between the two-photon entanglement and four-photon frustrated interference is twofold:

1. From the space-time configuration perspective: While the four-photon frustrated interference expands the source into a larger space by swapping the idlers, the phase-setting event **a** (phase α) can be space-like separated from the detection event **B**. We call this non-local quantum interference (Supplementary Fig. 9c below). This situation for the four-photon frustrated interference is similar to the two-photon entanglement (Supplementary Fig. 9a below), in which the setting events **a**/**b** can be space-like separated from the detection events **B**/**A**.

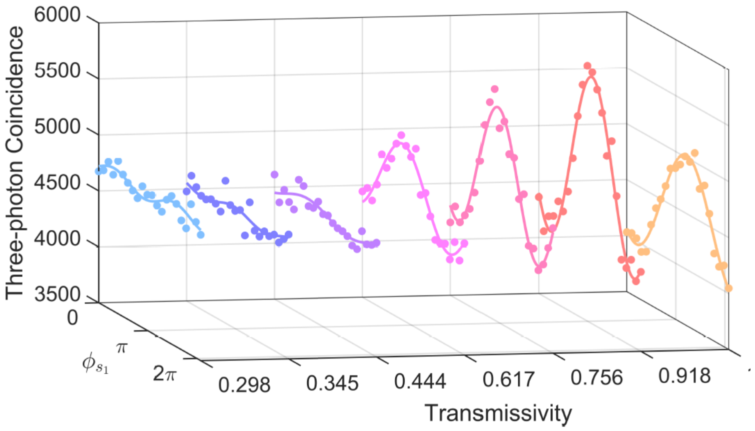

Supplementary Fig. 8. The relationship between the visibility of three-photon coincidence and the transmissivity of photon s2.

2. From the experimental observation perspective: For an EPR state (Supplementary Fig. 9a below), when Alice and Bob change their measurement settings, their single counts remain constant, while the coincidence between them shows dependence on both settings. In the four-photon frustrated interference (Supplementary Fig. 9c below), when Alice and Bob change their phase settings (α and β), their local two-photon coincidence counts (photons 1 and 2, photons 3 and 4, respectively) remain constant, while the four-photon coincidence shows the interference depending on both settings. In particular, when Alice and Bob set the phase $\alpha + \beta = \pi$ and keep them unchanged, there will be no four-photon coincidence counts. Therefore, when Alice detects two photons (event **A**), she can state that there must be no two-photon coincidence counts at Bob's side (event **B**); that is, event **A** prohibits event **B** with the phase setting $\alpha + \beta = \pi$, no matter how far Lab1 and Lab2 are separated from each other. We call this photon-count conditional probability between Alice and Bob the non-local quantum interference.

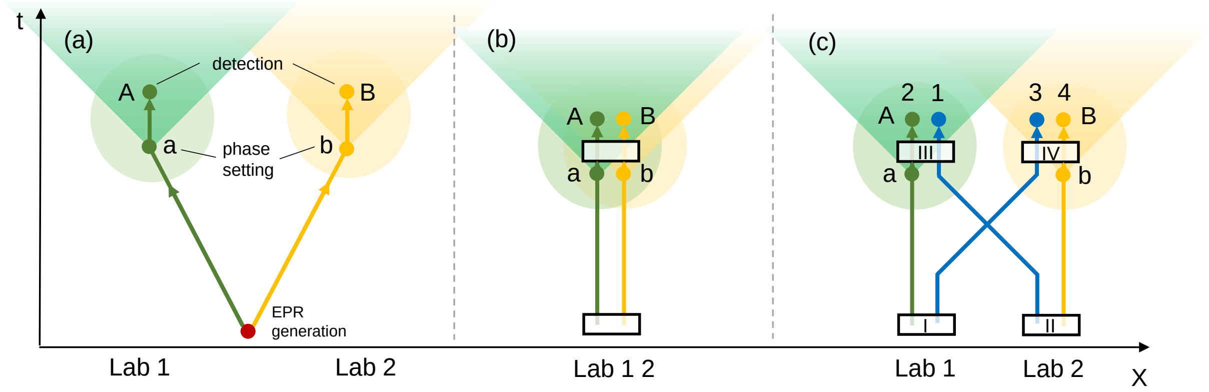

Supplementary Fig. 9. Space-time diagram of the three experiment settings. (a) Entangled state. (b) Two-photon frustrated interference. (c) Four-photon frustrated interference.

So far, in our experiment, a single laser is the pump source for all of the photon pair generations – and this could open the loophole at the time of the creation of the photon pairs in the lower layer, the phase setting is already fixed. However, this is only done for technical reasons. In principle, the phenomenon we have observed could be demonstrated under strict Einstein locality conditions[2,3]. There are several possible implementations, and we explain two of them now, referring to Fig. 1c:
(1) If the pump source is a pulsed laser, then the phases α and β can be set randomly after the pump pulse goes through the two crystals in the lower layer.
(2) The pump sources could be four independent phase-stable lasers. In that way, again it is possible to separate the four sources and the phases α and β, such that the phases are set only after the photon pairs are produced in the lower layer.

Note this requires that the phase of Alice and Bob should be in the same reference frame of the pump light, which

may potentially open certain loopholes, such as the freedom-of-choice loophole, especially when one consider the deterministic model. See details in [PNAS 107, 19708 (2010)][4]. However, to close all loopholes is beyond the scope of the current work and we plan to address this interesting topic in the future. Additionally, investigating the relations of these experimental design to other loopholes will be an interesting theoretical study[5]. To close the locality loophole, we can introduce a random phase on the pump, and configure the phase setting event for Alice/Bob outside the light cone of the phase setting event for the pump.

Supplementary Note 8 – Cancel the noise of three-photon interference

Here we give an example of source configuration to improve the three-photon interference visibility. In the main text, it is shown that the maximal three-photon interference visibility is 50% because of the constant noise term $|1,0,1,2\rangle$ produced by a simultaneous event in crystals II and IV.

This, however, is not a fundamental limit, as the additional noise contribution can be canceled by destructive interference (Supplementary Fig. 10). Let's first understand this via the graph representation of our experiment. As shown in Fig. S10a below, each vertex represents the path mode of photons. Each blue edge represents a photon pair source, (i.e., a non-linear crystal), which is labeled from I to IV as shown in Fig. 2. The noise term reducing the three-photon interference (photons 1, 3, and 4 in our experiment) is $|1,0,1,2\rangle$. So we have to make revisions in the setup in modes 1, 3, and 4. We now add four more crystals: V, VI, VII, and VIII. Crystal V emits non-collinear pairs into modes 1 and 3. Crystal VI produces two photons collinearly in path 4. They together produce an additional three-fold detection in the form $|1,0,1,2\rangle$. If we set the phase of the light that pumps crystal VI to π with respect to the light that pumps crystal I, then the noise contribution cancels with the newly created term.

At this stage, two additional terms emerge from a combination of crystal V with crystal II and with crystal IV. However, those contributions can also be canceled by adding new crystals VII and VIII that produce two photons collinearly in paths 1 and 3, respectively. Therefore, all noise contributions cancel and the resulting three-photon interference has 100% visibility. Detailed terms (on modes 1, 3, 4) generated from newly added crystals are listed below:

Noise cancel: Crystal V & VI: $-|1,0,1,2\rangle$

Newly created noise terms that have coincidence count:

Crystal V & II: $|2,0,1,1\rangle$

Crystal V & IV: $|1,0,2,1\rangle$

Terms from the contribution of Crystals VII and VIII:

Crystal VI & VII: $-|2,0,1,1\rangle$

Crystal II & VIII: $-|1,0,2,1\rangle$

The four above terms cancel.

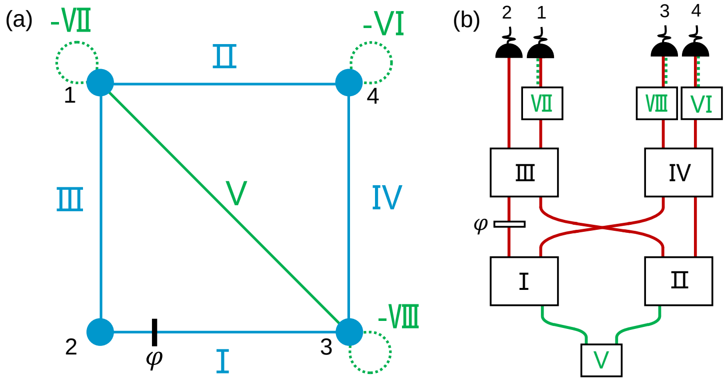

Supplementary Fig. 10. (a) Graph representation of noise cancellation. (b) The noise of three-photon interference can be completely restrained by adding 4 crystals marked in green.

Supplementary Note 9 – The Cause of non-local quantum interference

As shown in Supplementary Fig. 11, one can generate the GHZ state from a system similar to that of the frustrated four-photon interference, except that we use mode shifters to convert the photons coming from crystals I and II

from state $|0000\rangle$ to $|1111\rangle$. In this configuration, the GHZ state $|\Psi_{GHZ}\rangle = \frac{1}{\sqrt{2}}[|1111\rangle + e^{i\varphi}|0000\rangle]$ will not show interference when one traces over the undetected photon (photon 4), as the photons 123 are in a mixed state $\rho_{123} = \frac{1}{2}|000\rangle\langle 000| + \frac{1}{2}|111\rangle\langle 111|$. However, when we remove the mode shifter of the undetected photon (photon 4), the undetected photon remains in $|0\rangle$. The final state becomes a bipartite product state between particles 123 and 4: $|\Psi_{\text{product}}\rangle = \frac{1}{\sqrt{2}}[|111\rangle + e^{i\varphi}|000\rangle]|0\rangle$. As the state of photons 123 contains the phase information: $|\Psi\rangle_{123} = \frac{1}{\sqrt{2}}[|111\rangle + e^{i\varphi}|000\rangle]$, the interference shows up with the state $|\Psi_{\text{product}}\rangle$ even if we take the trace over the undetected photon (photon 4) and measure photons 1, 2, and 3 in mutually unbiased bases, for example in the $|+++\rangle$ base. Therefore, in this case the non-local quantum interference with undetected photon comes from the path identity rather than entanglement. Only when there are identical modes of the undetected photon, will the phase information (φ) on the undetected photon be transferred to the other three photons.

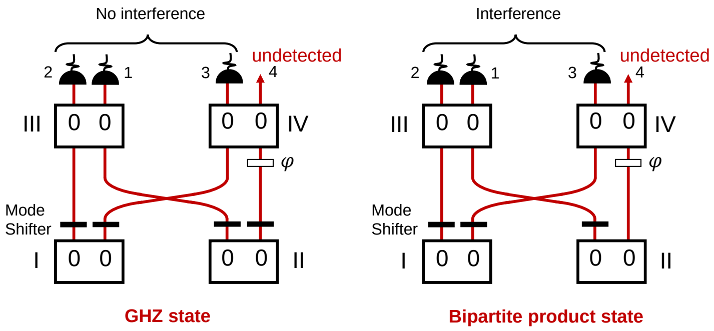

Supplementary Fig. 11. The interference with an undetected photon of the GHZ state and the bipartite product state.

REFERENCES

---

* These two authors contributed equally to this work

† These two authors contributed equally to this work; kai.wang@nju.edu.cn

‡ mario.krenn@mpl.mpg.de

§ Xiaosong.Ma@nju.edu.cn

1 Pittman, T. B. *et al.* Can two-photon interference be considered the interference of two photons? *Phys. Rev. Lett.* **77**, 1917–1920 (1996).

2 Gu, X., Erhard, M., Zeilinger, A. & Krenn, M. Quantum experiments and graphs ii: Quantum interference, computation, and state generation. *Proc. Nat. Acad. Sci.* **116**, 4147–4155 (2019).

3 Feng, L.-T. *et al.* On-chip quantum interference between the origins of a multi-photon state. *Optica* **10**, 105–109 (2023).

4 Scheidl, T. *et al.* Violation of local realism with freedom of choice. *Proc. Nat. Acad. Sci.* **107**, 19708–19713 (2010).

5 Larsson, J.-Å. Loopholes in bell inequality tests of local realism. *J. Phys. A Math. Theor.* **47**, 424003 (2014).
